# Vitamin B12 and Progression of White Matter Lesions. A 2-Year Follow-Up Study in First-Ever Lacunar Stroke Patients 

**DOI:** 10.1371/journal.pone.0078100

**Published:** 2013-10-14

**Authors:** Ellen C. van Overbeek, Julie Staals, Robert J. van Oostenbrugge

**Affiliations:** 1 Department of Neurology, Maastricht University Medical Centre, Maastricht, The Netherlands; 2 Cardiovascular Research Institute Maastricht (CARIM), Maastricht University Medical Centre, Maastricht, The Netherlands; University of Cambridge, United Kingdom

## Abstract

In cross-sectional studies periventricular white matter lesions (WML) were related to low plasma levels of vitamin B12. Whether low vitamin B12 levels are also related to progression of WML is still unknown. We studied baseline vitamin B12 levels and its association with progression of WML over 2 years of follow-up in first-ever lacunar stroke patients. In 107 first-ever lacunar stroke patients in whom baseline brain MRI and vitamin B12 status were available, we obtained a follow-up brain MRI after 2 years. We assessed progression of periventricular WML (pWML) and deep WML (dWML) using a visual WML change scale. We studied the relationship between baseline levels of plasma vitamin B12 and progression of WML after 2 years of follow-up by binary logistic regression analyses. Vitamin B12 deficiency was more frequent in patients with progression of pWML compared to those without progression (41.9% and 19.7% respectively, p = 0.02). Corrected for sex and age, progression of pWML was associated with lower baseline levels of vitamin B12 (OR 1.42 per 50 unit decrease, 95% CI 1.00-1.92). Vitamin B12 levels were not associated with progression of dWML. In conclusion progression of pWML after 2 years of follow-up relates to low levels of vitamin B12 at baseline in first-ever lacunar stroke patients. Whether this population could benefit from vitamin B12 supplementation is unknown and requires further investigation.

## Introduction

Lacunar infarcts and white matter lesions (WML) are common features of cerebral small vessel disease (cSVD) [1,2]. Derangement of the blood-brain barrier is thought to be an important underlying initiating cause of cSVD [1,2]. One of the factors that may play a role in maintaining the integrity of the blood-brain barrier is vitamin B12 [3-5]. 

In a cross-sectional population-based study vitamin B12 status was associated with severity of periventricular WML (pWML) and, to a lesser extent, also with deep WML (dWML) [6]. Furthermore, we found pWML to be related to low vitamin B12 levels in patients with small vessel (i.e. lacunar) stroke [7]. However the association between vitamin B12 and WML has not yet been confirmed in longitudinal studies. Progression of WML is associated with gait abnormalities, cognitive impairment and urinary disturbances, and therefore it could be important to identify associated factors that may be treatable [8,9].

The aim of this study was to determine whether low levels of vitamin B12 are related to progression of WML over 2 years of follow-up in lacunar stroke patients to further confirm a cause-and-effect relationship. 

## Methods

### Ethics statement

Data were collected from a substudy of an ongoing lacunar stroke project that was approved by the local medical ethical committee (METC, Maastricht). All patients gave written informed consent. 

### Patients

At baseline we included 124 first-ever lacunar stroke patients of whom vitamin B12 levels and brain MRI were available, as described before [7]. We defined lacunar stroke as an acute stroke syndrome with a compatible, small, deep infarct on MRI. In case MR imaging showed no symptomatic lesion, lacunar stroke was defined using the established criteria of specific lacunar syndromes [10]. Patients with potential cardiac embolic source of stroke or carotid stenosis were not included. At baseline vascular risk factors such as age, sex, diabetes mellitus, current smoking, total cholesterol level > 5.0 mmol/l and hypertension (based on patients medical history at admission) were recorded. All patients were offered a clinical follow-up MRI after 2 years as well as 24-hour ambulatory blood pressure monitoring.

### MRI scoring

MR images at baseline and follow-up were obtained with a 1.5 Tesla (T) or 3 T MR scanner (Philips) and consisted of axial T2 weighted and FLAIR sequence with fixed scanning parameters that were unchanged between baseline and follow-up. A symptomatic lacunar infarct was defined as a hyperintense lesion of < 20 mm in diameter on T2 and FLAIR images, with its site compatible to the clinical syndrome. DWI was not part of the scan protocol at that time. Asymptomatic lacunar infarcts were defined as hyperintense lesions on T2 images with corresponding hypo-intense lesions with a hyperintense rim on FLAIR and a diameter of < 20 mm. Baseline MRI was scored for pWML and dWML using the Fazekas scale [11]. We defined the presence of pWML as Fazekas scale 3 (irregular periventricular hyperintensities extending into the deep white matter). The presence of dWML was defined as (early) confluent deep WML, Fazekas scale 2 or 3. These Fazekas scores are histopathologically related to cerebral SVD [12,13]. 

WML progression on follow-up MRI was scored using the WML change scale as proposed by Prins et al [14]. This scale scores white matter changes in three periventricular regions (frontal caps, lateral bands and occipital caps) and four deep regions (frontal, parietal, temporal, occipital). Increase of WML was defined as new lesions or increase of existing lesions. In each region we assessed whether there was an increase in WML (+1), or no change in WML (0). Although not seen in our cohort, a decrease in WML (-1) could also be scored, adding up to a total score ranging from -3 to +3 in periventricular regions and -4 to +4 in deep regions. Progression was defined as a total periventricular score ≥ 1 or a total deep score ≥ 1 [14]. The WML progression was assessed by two vascular neurologists, who were blinded for vitamin B12 status, independently. The inter-rater agreement for total progression was substantial (ĸ 0.62). In case of disagreement a consensus meeting was held. 

### Vitamin B12

Blood samples were taken within 3 months after stroke. Plasma levels of vitamin B12 at baseline were assayed using a solid-phase time-resolved fluoroimmunoassay on an Auto Delfia immunoanalyser (PerkinElmer) as described elsewhere [7]. Vitamin B12 level > 150 pmol/L was defined as normal [7].

### Statistical analysis

Statistical analysis was performed using PASW Statistics 18. Differences between groups were determined using χ^2^-test, *t* test, or Mann–Whitney test, where appropriate. We assessed the relationship between low levels of plasma vitamin B12 at baseline (independent factor) and progression of WML at 2-years follow-up (dependent factor) by binary logistic regression analysis adjusted for age and sex. Furthermore we performed additional exploratory analyses in which we adjusted for cardiovascular risk factors (hypertension, diabetes mellitus, hypercholesterolemia and current smoking), 24-hour mean arterial pressure (MAP) at follow-up and presence of extensive deep or periventricular WML at baseline by adding them one at a time to the model. Odds ratios (OR) are given with 95% confidence interval (CI). Statistical significance was considered at *P* < 0.05.

## Results

### Patients

Of 124 included first-ever lacunar stroke patients at baseline, a 2-year follow-up MRI (mean 24.9 ± 1,8 months) was obtained in 110 as 3 patients declined a follow-up MRI, 2 patients died (of non-neurological cause) and 9 patients were lost to follow-up. Another 3 patients were excluded because of inadequate scan data, leaving 107 (86.3%) patients for further analysis. Characteristics of patients who were not included in follow-up were comparable to those of the included patients concerning age, sex and baseline vitamin B12 levels. Extensive WML (both pWML and dWML) at baseline were slightly more frequent in patients who were not included. Eighty-seven patients were scanned at an 1.5 T MRI scanner both at baseline and follow-up. Baseline MR was performed at a 3 T scanner in 20 patients, and at follow-up 18 of these patients were scanned at an 1.5 T scanner and 2 patients were scanned at a 3 T scanner. 

Baseline characteristics of all 107 patients are presented in table 1. Mean vitamin B12 level was 208 pmol/L (± 82) and 28 patients (26.2%) had vitamin B12 level < 150pmol/L. Patients with low levels of vitamin B12 did not routinely receive supplementation, however 2 patients received treatment sometime within the 2-year follow-up period, resulting in vitamin B12 levels above 300 pmol/L. Thirtyone patients (29.0%) had extensive pWML (Fazekas 3) at baseline and 33 patients (30.8%) had extensive dWML (Fazekas 2 + 3) at baseline. 

**Table 1:** Baseline characteristics.

	All patients N = 107	Periventricular WML progression N = 31	No periventricular WML progression N =76	P value	Deep WML progression N = 42	No deep WML progression N = 65	P value
Male	62 (57.9%)	18 (58.1%)	48 (63.2%)	0.62	22 (52.4%)	44 (67.7%)	0.11
Age (years)	63.5 (± 11.8)	69.2 (± 8.5)	61.1 (± 12.2)	< 0.01	68.0 (± 9.7)	60.5 (± 12.2)	< 0.01
Vitamin B12 (pmol/L)	208.0 (± 82.2)	182.6 (± 75.2)	218.3 (± 83.1)	0.04	206.4 (± 103.6)	209.0 (± 65.5)	0.87
Vitamin B12 <150 pmol/L	28 (26.2%)	13 (41.9%)	15 (19.7%)	0.02	13 (31.0%)	15 (23.1%)	0.37
Extensive periventricular WML at baseline (Fazekas 3)	31 (29.0%)	18 (58.1%)	13 (17.1%)	< 0.01	19 (45.2%)	12 (18.5%)	< 0.01
Extensive deep WML at baseline (Fazekas 2 + 3)	33 (30.8%)	17 (54.8%)	16 (21.1%)	< 0.01	25 (59.5%)	8 (12.3%)	< 0.01
Hypertension	71 (66.4%)	21 (67.7%)	50 (65.8%)	0.85	30 (71.4%)	41 (63.1%)	0.37
Diabetes Mellitus	16 (15.0%)	5 (16.1%)	11 (14.5%)	0.78	8 (19.0%)	8 (12.3%)	0.34
Hypercholestrolemia	79 (73.8%)	26 (83.9%)	53 (69.7%)	0.13	32 (76.2%)	47 (72.3%)	0.66
Current smoking	48 (44.9%)	14 (44.2%)	34 (44.7%)	0.97	15 (35.7%)	33 (50.8%)	0.13
24-hour mean arterial pressure (mmHg) at follow-up*	102.1 (± 9.7)	104.2 (± 9.0)	101.2 (± 9.8)	0.15	101.1 (± 9.1)	102.7 (±10.0)	0.44

WML: white matter lesions; * 6 missing data

### Progression of WML

Progression of pWML was seen in 31 patients (29.0%) and progression of dWML was seen in 42 patients (39.3%). Patients with WML progression at follow-up were significantly older compared to patients with no progression (68.1 ± 9.5 vs. 59.1 ± 12.2 years old, p < 0.01) . In patients with progression of pWML at follow-up extensive pWML (fazekas 3) at baseline was more frequent compared to patients without pWML progression (18 (58.1%) vs. 13 (17.1%), p < 0.01). Also in patients with progression of dWML, extensive dWML (fazekas 2 + 3) at baseline was more frequent compared to patients without dWML (25 (59.5%) vs. 8 (12.3%), p < 0.01). At follow-up, blood pressure monitoring was performed in 101 patients (94.4 %). 24-hours MAP values were not significantly higher in patients with pWML or dWML progression (table 1).

### Vitamin B12 and progression of WML

Vitamin B12 plasma levels were significantly lower in patients who showed progression of pWML and these patients also had more often vitamin B12 level <150 pmol/L (table 1). Vitamin B12 levels did not differ between patients with or without dWML progression. 

In table 2 we present the results of logistic regression analyses. Lower baseline levels of plasma vitamin B12 were associated with progression of pWML (OR 1.42 (per 50 pmol/L decrease in vitamin B12 level), 95% CI 1.00-1.92). This association remained significant after correction for sex and age (OR 1.42 (per 50 pmol/L decrease in vitamin B12 level), 95% CI 1.05-2.02). Adding cardiovascular risk factors (not shown), 24-hour MAP at follow-up (OR 1.49, (per 50 pmol/L decrease in vitamin B12 level) 95% CI 1.11-2.13) and baseline presence of extensive pWML (OR 1.49, (per 50 pmol/L decrease in vitamin B12 level) 95% CI 1.05-2.13) to the model one at a time provided similar results. 

**Table 2:** Binary logistic regression analysis with progression of periventricular white matter lesions as dependent factor.

	Progression of periventricular white matter lesions		
	Model 1: Unadjusted, OR (95% CI)	Model 2: Adjusted for sex and age, OR (95% CI)	Model 3: Adjusted for sex, age and baseline pWML, OR (95% CI)
Vitamin B 12 level (pmol/L decrease)	1.01 (1.00-1.01)*	1.01 (1.00 -1.01)*	1.01 (1.00 -1.02)*
Vitamin B12 level (50 pmol/L decrease)	1.42 ( 1.00-1.92)*	1.42 (1.05-2.02)*	1.49 (1.05-2.13)*
Vitamin B12 <150 pmol/L	2.94 (1.18-7.30)*	3.05 (1.16-8.24)*	2.56 (1.02-8.06)*

* p < 0.05

Vitamin B12 deficiency < 150 pmol/L was also associated with progression of pWML (OR 2.94, 95% CI: 1.18-7.30). This association also remained after correction for sex and age (OR 3.05, 95% 1.16-8.24) and after adding cardiovascular risk factors (not shown), 24-hour MAP at follow-up (OR 2.89, 95% CI 1.03-8.09) and baseline presence of extensive pWML (OR 2.56, 95% CI 1.02-8.06) to the model. Progression of dWML was not associated with absolute vitamin B12 level, nor with vitamin B12 < 150pmol/L. 

## Discussion

We found that low baseline plasma levels of vitamin B12 relate to progression of pWML, but not dWML, after 2 years of follow-up in patients with first-ever lacunar stroke. 

Previous cross-sectional studies already showed that the severity of pWML, was related to low vitamin B12 levels, whereas dWML were not [6,7]. These studies were done in patients with lacunar stroke and in a healthy population [6,7]. Our study is the first longitudinal study that explored the association between vitamin B12 level and progression of WML and confirms the association between pWML and vitamin B12. 

The different results for pWML en dWML are intriguing. pWML are thought to differ from dWML in MRI appearance, underlying neuropathology and vascular supply [12,13]. Differences between pWML and dWML were also found concerning genetic and vascular risk factors, and progression rate [12,13]. This supports the idea that differences in pathophysiology may be present and seemingly the role of risk factors such as vitamin B12 may differ. It should be said however that the relatively small study sample size and the rather low prevalence of vitamin B12 deficiency in our study can not definitely rule out an association between low vitamin B12 levels and dWML and the separation between deep and periventricular WML still remains controversial.

The precise actions of vitamin B12 in the brain and in the pathophysiology of cerebral WML still need to be illuminated. Effects of vitamin B12 are complex and mostly attributed to its role in lowering homocysteine levels [15-17]. Hyperhomocysteinemia is related to both endothelial dysfunction and WML [15,16]. Unfortunately, we did not measure levels of homocysteine in our study. Nevertheless vitamin B12 also seems to effect the white matter beyond the homocysteine lowering effect [3,18]. It has been hypothesized that vitamin B12 directly affects myelin structure and function [3,6,18]. It may also be that vitamin B12 affects the integrity of the blood-brain barrier. Dysfunction of the blood-brain barrier is the most recent theory on the cause of cSVD-related WML [1,2]. Although structural BBB derangement has never been proven in vitamin B12 deficiency, it is possible that BBB permeability is increased at a functional level; while structural integrity is retained [3]. In vitamin B12 deficient rats increased TNF-alpha and decreased IL-6 levels in the central nervous system have been found [3,4]. TNF-alpha increases blood-brain barrier permeability [19]. In humans the cerebrospinal fluid / serum albumin ratio, which is considered to reflect the permeability of the blood-brain barrier, was decreased in patients treated with vitamin B12-B6-folate combination [5].

Our results imply that patients with cSVD could benefit from supplementation of vitamin B12 to prevent further progression of WML over time. In a subgroup analysis of the VISP trial it was found that B-vitamin therapy improved survival free of cardiovascular events in those patients with a non disabling stroke who were more likely to respond to vitamin B12-supplementation (namely excluding those likely to have vitamin B12 malabsorption, those who received parenteral vitamin B12 and other vitamin B12 supplements, and those with renal failure)[20]. A subgroup analysis of the VITATOPS trial, that studied the effect of B-vitamin in patients with recent stroke or TIA, suggested that vitamin B12 supplementation might reduce the risk of stroke in patients with lacunar stroke [21]. In another substudy of the VITATOPS trial that assessed progression of WML in patients with recent stroke or TIA, only those patients with severe cSVD (that is in patients with a Fazekas score of dWML ≥ 2 and (acute or old) lacunes) at baseline, benefited from B-vitamin supplementation in terms that WML progression was reduced [22]. Within the VITATOPS trial however all patients received B-vitamin supplementation irrespectively of the initial serum levels of these vitamins. One might expect a greater effect of B-vitamin supplementation in patients with low levels of vitamin B12. Furthermore, higher dosage than used in both trials might be needed for optimal effects. In our study, patients with low levels of vitamin B12 did not routinely receive supplementation. Two patients with baseline levels of 102 pmol/L and 635 pmol/L respectively, received treatment sometime within the 2-year follow-up period. Despite treatment, both patients showed progression of WML over time.

Additional studies are needed to investigate whether supplementation of vitamin B12 may reduce progression of WML in lacunar stroke patients with low levels of vitamin B12. 

We found a rather high prevalence of vitamin B12 deficiency of 26.2% in our study population compared to previously reported prevalences, although ranges of 12 to 30% have been mentioned [23]. Also one cannot rule out that vitamin B12 deficiency is more frequent in a population of patients with symptomatic small vessel disease compared to general stroke populations or community based populations.

In a general population study, high blood pressure levels preceded WML progression and therefore blood pressure might be a confounder in the association between vitamin B12 and WML progression [24]. We adjusted our data for 24-hour MAP at follow-up which did not alter our results. This further strengthens our conclusions. However, it has to be said that adjusting for blood pressure levels at follow-up does not account for other factors such as blood pressure levels in the past, duration of hypertension, different drug classes that were used and changes in blood pressure levels.

Among the strengths of our study is a well-defined population of first-ever lacunar stroke patients. Considering the strict inclusion criteria this study was conducted in a reasonable sized population. However our study also has limitations. First, vitamin B12 was only measured once at baseline. Vitamin B12 levels may have altered over time and we can not exclude that patients used over-the-counter vitamins. Secondly, plasma vitamin B12 may not be the most accurate marker to identify low intracellular concentrations of vitamin B12. Markers of vitamin B12 status are methyl malonic acid (MMA), total homocysteine (tHcy) and holotranscobalamin (HoloTC), which tests were not routinely performed in our study. However, in studies, using elevated levels of MMA as a gold standard, it has been shown that plasma vitamin B12 levels were adequate for identifying patients with functional vitamin B12 deficiency [25]. One might consider differences in field strength of MRI (1.5T and 3T) to be a third limitation. In 89 patients, baseline and follow-up scans were performed on the same type of scanner (87 patients had 1.5T MRI at both baseline and follow-up, 2 patients had 3T at baseline and follow-up). In 18 patients we compared 3T baseline scan with a 1.5T follow-up scan. In these patients WML progression could have been underestimated but not overestimated. However, Wardlaw et al. found no objective evidence that lesion detection is increased in 3T, and furthermore, repeating our main analysis in those 87 patients with both scans at 1.5T yielded largely unchanged conclusions (results not shown) [26]. Fourthly we assessed the progression of WML using a visual scale. Volumetric measurements were not available. The visual WML change scale we used was found to be highly correlated to changes in white matter hyperintensities volume and it is the best alternative visual scale for measuring WML progression [14,27]. Furthermore Diffusion-Weighted Imaging (DWI) was not routinely included in our baseline MRI protocol, so in some of our patients we could not identify or could have wrongly identified the symptomatic lacunar infarct. However, the strict clinical criteria for lacunar syndromes minimize the chance that we wrongly included a lacunar stroke. Finally we did not include any clinical outcome measure, but this was beyond the aim of our study. A larger scale, prospective study is needed to further confirm the clinical relevance of the association of progression of WML and vitamin B12. 

In conclusion, progression of pWML at 2-years follow-up relates to low levels of vitamin B12 at baseline in first-ever lacunar stroke patients. Whether this well-defined population could benefit from vitamin B12 supplementation is yet unknown and requires further investigation. 
